# Vitamin D3 inhibits lipopolysaccharide-induced placental inflammation through reinforcing interaction between vitamin D receptor and nuclear factor kappa B p65 subunit

**DOI:** 10.1038/srep10871

**Published:** 2015-06-12

**Authors:** Yuan-Hua Chen, Zhen Yu, Lin Fu, Hua Wang, Xue Chen, Cheng Zhang, Zheng-Mei Lv, De-Xiang Xu

**Affiliations:** 1 School of Public Health, Anhui Medical University, Hefei, China; 2 Anhui Provincial Key Laboratory of Population Health & Aristogenics, Anhui Medical University, Hefei, China; 3 School of Basic Medical Science, Anhui Medical University, Hefei, 230032, China

## Abstract

It is increasingly recognized that vitamin D3 (VitD3) has an anti-inflammatory activity. The present study investigated the effects of maternal VitD3 supplementation during pregnancy on LPS-induced placental inflammation and fetal intrauterine growth restriction (IUGR). All pregnant mice except controls were intraperitoneally injected with LPS (100 μg/kg) daily from gestational day (GD)15–17. In VitD3 + LPS group, pregnant mice were orally administered with VitD3 (25 μg/kg) before LPS injection. As expected, maternal LPS exposure caused placental inflammation and fetal IUGR. Interestingly, pretreatment with VitD3 repressed placental inflammation and protected against LPS-induced fetal IUGR. Further analysis showed that pretreatment with VitD3, which activated placental vitamin D receptor (VDR) signaling, specifically suppressed LPS-induced activation of nuclear factor kappa B (NF-κB) and significantly blocked nuclear translocation of NF-κB p65 subunit in trophoblast gaint cells of the labyrinth layer. Conversely, LPS, which activated placental NF-κB signaling, suppressed placental VDR activation and its target gene expression. Moreover, VitD3 reinforced physical interaction between placental VDR and NF-κB p65 subunit. The further study demonstrates that VitD3 inhibits placental NF-κB signaling in VDR-dependent manner. These results provide a mechanistic explanation for VitD3-mediated anti-inflammatory activity. Overall, the present study provides evidence for roles of VDR as a key regulator of placental inflammation.

Lipopolysaccharide (LPS) is a toxic component of cell walls in gram-negative bacteria and is widely present in the digestive tracts of humans and animals^1^. Humans are constantly exposed to low levels of LPS through infection. Gastrointestinal inflammation and excess alcohol intake elevate permeability of LPS from gastrointestinal tract into circulation^2^. Mimicking maternal infection by exposing pregnant rodents to LPS at different gestational stages has been associated with adverse pregnant outcomes. According to an earlier report, maternal LPS exposure at early gestational stage caused embryonic resorption^3^. Our recent reports showed that maternal LPS exposure at middle gestational stage caused neural tube defects^4^,^5^,^6^. We and others found that maternal LPS exposure at late gestational stages led to fetal demise, intra-uterine growth restriction (IUGR), skeletal development retardation, and preterm delivery^7^,^8^,^9^,^10^,^11^,^12^.

Increasing evidence demonstrates that inflammatory cytokines, such as tumor necrosis factor alpha (TNF-α) and interleukin (IL)-8, have been associated with LPS-induced adverse developmental outcomes^13^,^14^. Several reports showed that maternal LPS exposure during pregnancy elevated the levels of inflammatory cytokines in maternal serum, amniotic fluid, fetal liver and fetal brain^15^,^16^. TNF-α inhibitor prevented LPS-induced fetal IUGR and demise^17^. Moreover, chemokine inhibitor protected mice from LPS-induced preterm delivery^14^. Thus, anti-inflammation is an important strategy for the prevention of LPS-induced developmental impairment.

Vitamin D, a secosteroid hormone, is known for its classical functions in calcium uptake and bone metabolism^18^. Recently, vitamin D is recognized for its non-classical actions including the modulation of innate immune and the regulation of cell proliferation^19^,^20^. Indeed, vitamin D is a potent anti-inflammatory agent^21^,^22^,^23^. Vitamin D itself is devoid of biological activity. The actions of vitamin D are mediated by vitamin D receptor (VDR) that binds 1,25(OH)2D3, the active form of vitamin D, to induce both transcriptional and non-genomic responses^24^,^25^. Some reports demonstrate that vitamin D receptor (VDR) negatively regulates bacterial-induced intestinal NF-kappaB activation and attenuates response to infection^26^,^27^,^28^. Another study showed that VDR block TNFα-induced NF-κB activation and IL-6 up-regulation in HEK293 and RAW264.7 cells^29^.

The objective of the present study was to investigate the effects of pretreatment with vitamin D3 (VitD3) on LPS-induced placental inflammation and fetal IUGR in mice. Our results indicate that pretreatment with VitD3 protects against LPS-induced fetal IUGR through its anti-inflammatory activity. We demonstrate for the first time that VitD3 inhibits LPS-induced placental inflammation through reinforcing the interaction between VDR and NF-κB p65 subunit. We provide evidence for roles of VDR as a key regulator of placental inflammation.

## Results

### VitD3 prevents LPS-induced fetal IUGR and demise

**Figure 1:**
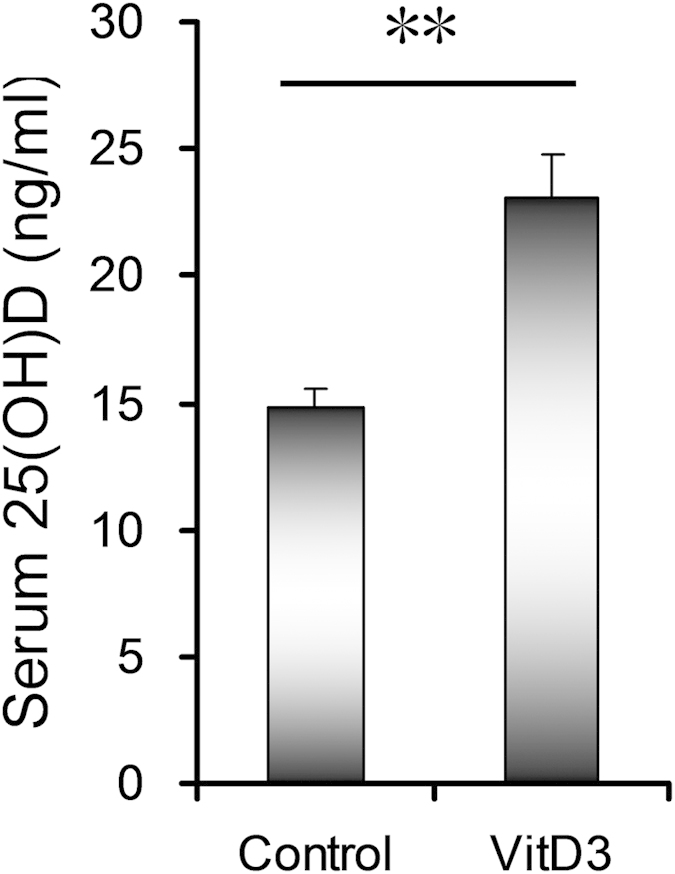
Maternal VitD3 supplementation increase serum 25(OH)D levels. In VitD3 group, pregnant mice were orally administered with VitD3 (25 μg/kg) daily from GD14 to GD17. All animals were sacrificed on GD18. Non-fasting blood samples were collected. Serum 25(OH)D was measured by Radioimmunoassay (RIA). All data were expressed as means ± S.E.M (n = 15). ^**^P < 0.01.

To investigate the effects of VitD3 on LPS-induced IUGR, all pregnant mice except control and alone LPS group were orally administered with VitD3 (25 μg/kg) daily. As expected, maternal serum 25(OH)D level was significantly increased in VitD3 group (Fig. 1). Moreover, no dams died and no abortion was observed throughout the pregnancy. As shown in Table 1, the number of dead fetuses per litter was significantly elevated in LPS-treated mice. By contrast, the number of live fetuses per litter was significantly reduced in LPS group. In addition, fetal weight and crown-rump length were significantly reduced in LPS-treated mice (Table 1). Although VitD3 alone had no effect on fetal development, VitD3 pretreatment during pregnancy protected against LPS-induced fetal demise (Table 1). Moreover, pretreatment with VitD3 significantly attenuated LPS-induced IUGR (Table 1).

### VitD3 inhibits LPS-induced placental inflammation

**Figure 2:**
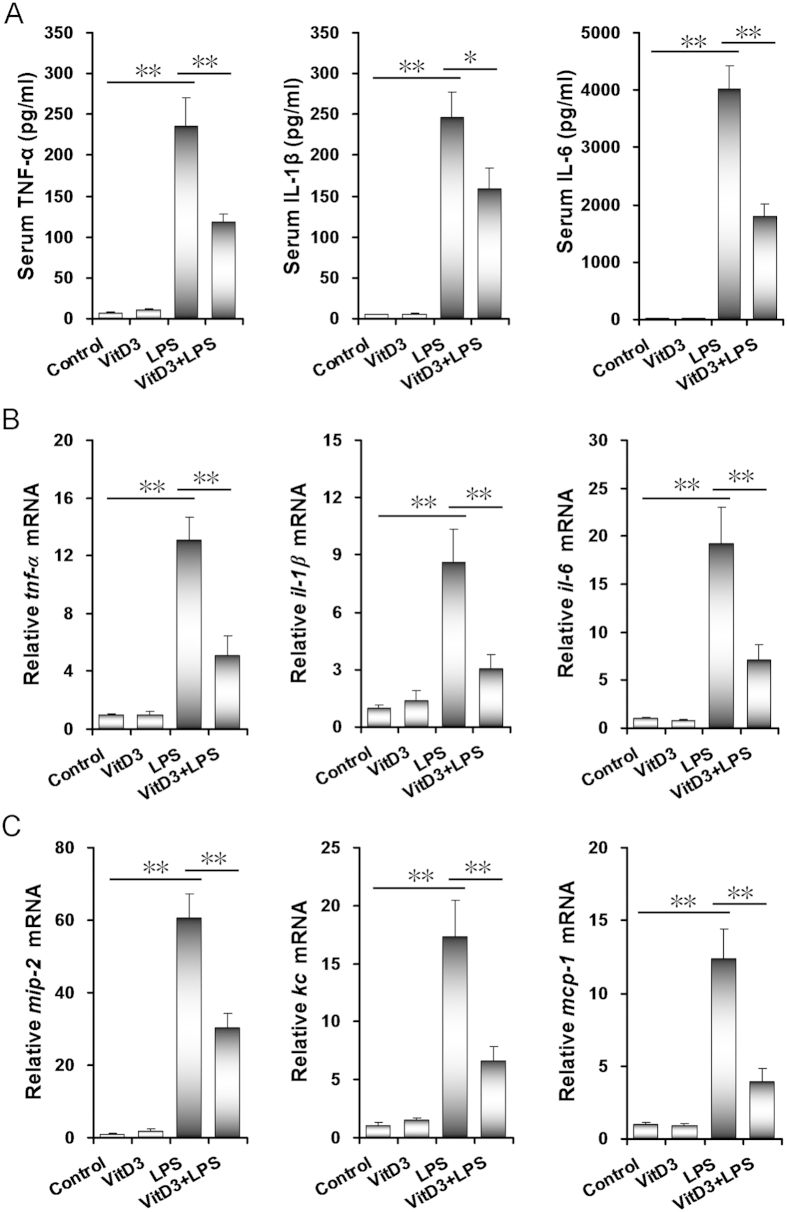
VitD3 alleviates on LPS-induced inflammatory cytokines. All pregnant mice were i.p. injected with LPS (100 μg/kg) on GD15. In VitD3 + LPS group, pregnant mice were pretreated with vitamin D3 (25 μg/kg) 24 h and 1 h before LPS injection. Maternal sera and placentas were collected 2 h after LPS injection. (**A**) Serum TNF-α, IL-1β and IL-6 were measured using ELISA. (**B**) Placental inflammatory cytokines and (**C**) chemokines were determined using real-time RT-PCR. All data were expressed as means ± S.E.M of six samples from six different pregnant mice. ^**^P < 0.01.

As expected, serum TNF-α, IL-1β and IL-6 levels were significantly elevated in LPS-treated dams. Interestingly, LPS-induced elevation of serum TNF-α, IL-1β and IL-6 was significantly attenuated in VitD3-pretreated pregnant mice (Fig. 2A). The effects of VitD3 on LPS-induced placental inflammatory cytokines and chemokines were then analyzed. As shown in Fig. 2B, mRNA levels of placental *tnf-α*, *il-1β* and *il-6*, three inflammatory cytokines, were obviously elevated in LPS group. Moreover, placental *mip-2*, *kc* and *mcp-1*, three chemokine genes, were markedly up-regulated in LPS-treated mice (Fig. 2C). Interestingly, LPS-induced up-regulation of placental inflammatory cytokines and chemokines was significantly attenuated in VitD3-pretreated mice (Fig. 2B,C).

### VitD3 does not inhibit LPS-activated placental MAPK and PI3K/Akt signaling

**Figure 3:**
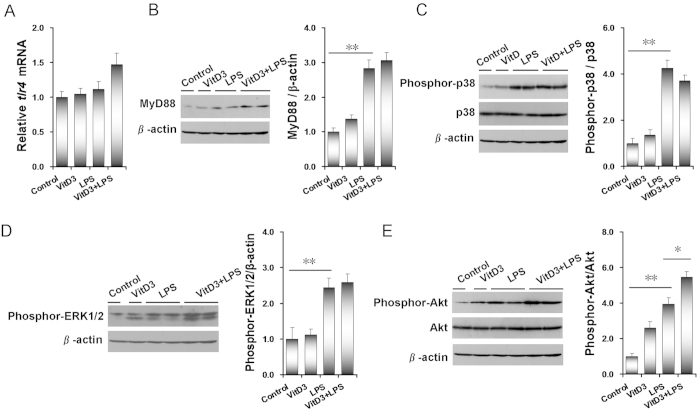
Effects of VitD3 on LPS-activated placental MAPK and PI3K/Akt signaling. All pregnant mice were i.p. injected with LPS (100 μg/kg) on GD15. In VitD3 + LPS group, pregnant mice were pretreated with vitamin D3 (25 μg/kg) 24 h and 1 h before LPS injection. Placentas were collected 2 h after LPS injection. (**A**) Placental *tlr4* mRNA was measured using real-time RT-PCR. (**B**–**E**) Placental MyD88, pp38, pERK1/2 and pAkt/Akt were measured using immunoblots. (**B**) MyD88. (**C**) pp38. (**D**) pERK1/2. (**E**) pAkt. All experiments were duplicated for four times. All data were expressed as means ± S.E.M. (n = 6–8). ^*^ *P* < 0.05, ^**^ *P* < 0.01.

As shown in Fig. 3A, no significant difference on placental *tlr4* mRNA was observed among different groups. Although placental MyD88 protein was significantly up-regulated in LPS-treated mice, VitD3 had little effect on LPS-induced up-regulation of placental MyD88 protein (Fig. 3B). The effects of VitD3 on LPS-activated placental p38 MAPK and ERK signaling are presented in Fig. 3C,D. As expected, placental pp38 and pERK1/2 were significantly elevated in LPS-treated mice. Unexpectedly, VitD3 had no effect on LPS-evoked placental p38 MAPK and ERK1/2 phosphorylation. The effects of VitD3 on LPS-induced placental Akt phosphorylation were then analyzed. As shown in Fig. 3E, placental pAkt was significantly elevated in LPS-treated mice. Unexpectedly, VitD3 did not inhibit LPS-evoked placental Akt phosphorylation. Actually, VitD3 slightly aggravated LPS-evoked placental Akt phosphorylation.

### VitD3 inhibits LPS-induced placental NF-κB activation

**Figure 4:**
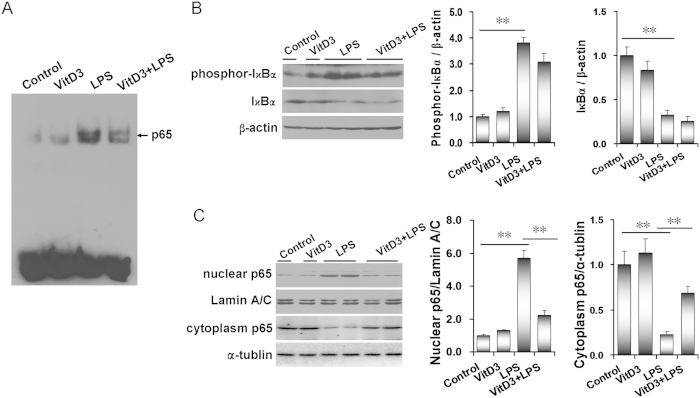
VitD3 inhibits LPS-induced placental NF-κB activation. All pregnant mice were i.p. injected with LPS (100 μg/kg) on GD15. In VitD3 + LPS group, pregnant mice were pretreated with vitamin D3 (25 μg/kg) 24 h and 1 h before LPS injection. Placentas were collected 2 h after LPS injection. (**A**) Placental NF-κB binding activity was detected using EMSA. (**B**-**C**) Placental phosphor-IκBα, IκBα and NF-κB p65 subunit were detected using immunoblots. (**B**) phosphor-IκBα and IκBα. (**C**) nuclear NF-κB p65 and cytoplasm NF-κB p65. All experiments were duplicated for four times. All data were expressed as means ± S.E.M. (n = 6–8). ^**^ *P* < 0.01.

**Figure 5:**
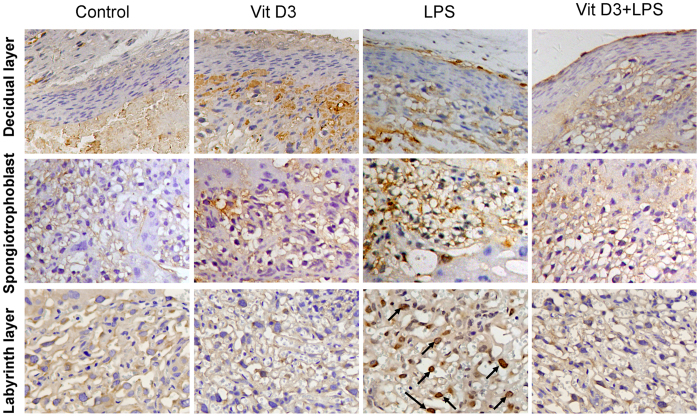
VitD3 inhibits LPS-induced nuclear translocation of placental NF-κB p65. All pregnant mice were i.p. injected with LPS (100 μg/kg) on GD15. In VitD3 + LPS group, pregnant mice were pretreated with vitamin D3 (25 μg/kg) 24 h and 1 h before LPS. Placentas were collected 2 h after LPS. Nuclear translocation of NF-κB p65 subunit was analyzed using immunohistochemistry. Representative photomicrographs of placental histology from mice treated with saline (control), VitD3, LPS and VitD3 + LPS are shown. Original magnification: 400×. Nuclear translocation of NF-κB p65 subunit was mainly distributed in trophoblast gaint cells of the labyrinth layer (arrows).

As expected, placental NF-κB binding activity was obviously elevated in LPS group (Fig. 4A). Further analysis showed that placental phosphorylated IκBα level was significantly increased in LPS-treated mice (Fig. 4B). Correspondingly, placental IκBα level was significantly decreased in LPS-treated mice (Fig. 4B). In addition, the level of placental nuclear NF-κB p65 subunit was markedly elevated in LPS group (Fig. 4C). Correspondingly, placental cytoplasm NF-κB p65 level was significantly decreased in LPS-treated mice (Fig. 4C). Interestingly, VitD3 significantly repressed LPS-evoked placental NF-κB binding activity (Fig. 4A). Although VitD3 had little effect on LPS-induced placental IκBα phosphorylation (Fig. 4B), pretreatment with VitD3 obviously attenuated LPS-induced placental translocation of NF-κB p65 subunit from cytoplasm to nuclei (Fig. 4C). The effects of VitD3 on LPS-induced nuclear translocation of NF-κB p65 subunit were further analyzed using IHC. As shown in Fig. 5, LPS-induced nuclear translocation of NF-κB p65 subunit was mainly distributed in trophoblast gaint cells of the labyrinth zone. Interestingly, VitD3 pretreatment almost completely blocked LPS-induced nuclear translocation of NF-κB p65 subunit in trophoblast gaint cells of the labyrinth zone (Fig. 5).

### LPS inhibits VitD3-activated placental VDR and its target genes

**Figure 6:**
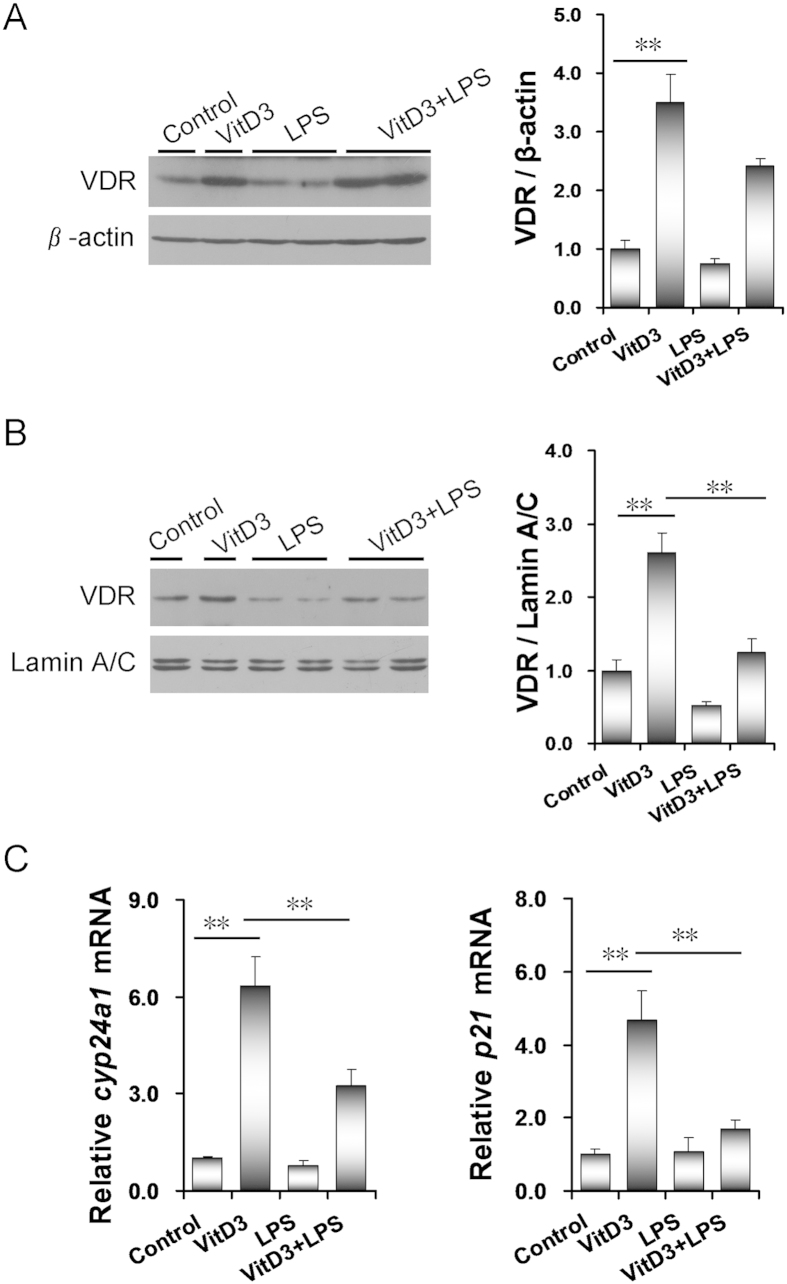
LPS inhibits VitD3-activated placental VDR signaling. All pregnant mice were i.p. injected with LPS (100 μg/kg) on GD15. In VitD3 + LPS group, pregnant mice were pretreated with vitamin D3 (25 μg/kg) 24 h and 1 h before LPS injection. Placentas were collected 2 h after LPS injection. (**A**) Placental VDR was detected using immunoblots. (**B**) Nuclear VDR was detected using immunoblots. All experiments were duplicated for four times. A representative gel for VDR (upper panel) and lamin A/C (lower panel) was shown. All data were expressed as means ± S.E.M. (n = 6–8). (**C**) Placental *cyp24a1* and *p21* mRNA was measured using real-time RT-PCR. All data were expressed as means ± S.E.M. (n = 8). ^**^ *P* < 0.01.

**Figure 7:**
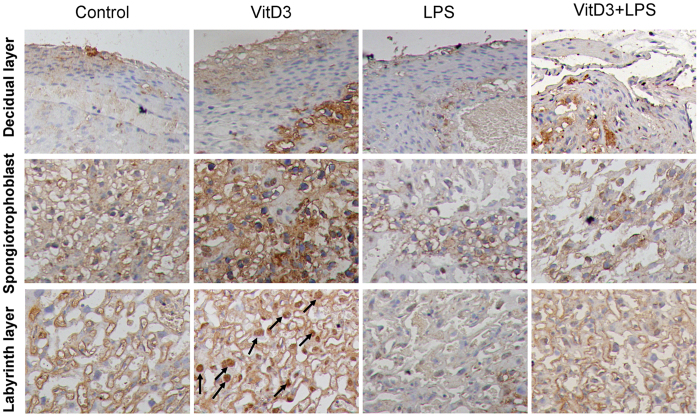
LPS inhibits VitD3-induced nuclear translocation of placental VDR. All pregnant mice were i.p. injected with LPS (100 μg/kg) on GD15. In VitD3 + LPS group, pregnant mice were pretreated with vitamin D3 (25 μg/kg) 24 h and 1 h before LPS. Placentas were collected 2 h after LPS. Nuclear translocation of VDR was analyzed using immunohistochemistry. Representative photomicrographs of placental histology from mice treated with saline (control), VitD3, LPS and VitD3 + LPS are shown. Original magnification: 400×. Nuclear translocation of VDR was mainly distributed in trophoblast gaint cells of the labyrinth layer (arrows).

As expected, placental VDR expression was significantly up-regulated in VitD3-pretreated mice (Fig. 6A). Moreover, placental nuclear VDR level was markedly elevated in VitD3-pretreated mice (Fig. 6B). Correspondingly, placental *Cyp24a1* and *p21,* two target genes of VDR signaling, were significantly up-regulated by VitD3 (Fig. 6C). Although LPS did not affect placental VDR expression (Fig. 6A), it markedly repressed placental translocation of VDR from cytoplasm to nuclei (Fig. 6B). In addition, LPS obviously repressed VitD3-induced up-regulation of placental *Cyp24a1* (Fig. 6C). The effects of LPS on VitD3-induced nuclear translocation of VDR were further analyzed using IHC. Consistent with LPS-induced nuclear translocation of NF-κB p65, VitD3-induced nuclear translocation of VDR was mainly observed in trophoblast gaint cells of the labyrinth layer (Fig. 7). Interestingly, LPS markedly inhibited VitD3-induced nuclear translocation of VDR in trophoblast gaint cells of the labyrinth zone (Fig. 7).

### VitD3 reinforces interaction between placental VDR and NF-κB p65 subunit

**Figure 8:**
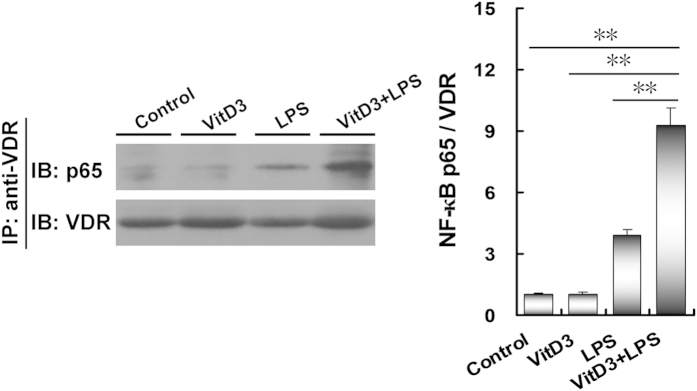
VitD3 reinforces interaction between placental VDR and NF-κB p65 subunit. All pregnant mice were i.p. injected with LPS (100 μg/kg) on GD15. In VitD3 + LPS group, pregnant mice were pretreated with vitamin D3 (25 μg/kg) 24 h and 1 h before LPS injection. Placentas were collected 2 h after LPS injection. The interaction between NF-κB p65 and VDR was detected using CoIP. All experiments were duplicated for four times. A representative gel for NF-κB p65 (upper panel) and VDR (lower panel) was shown. All data were expressed as means ± S.E.M. (n = 4). ^**^ *P* < 0.01.

The interaction between placental VDR and NF-κB p65 subunit was determined by CoIP. As shown in Fig. 8, treatment with VitD3 plus LPS markedly increased the level of NF-κB p65 in the immunocomplexes precipitated by anti-VDR antibody. These results suggest that VitD3 reinforces the interaction between placental VDR and NF-κB p65 subunit.

### VitD3 inhibits placental NF-κB signaling in VDR-dependent manner

**Figure 9:**
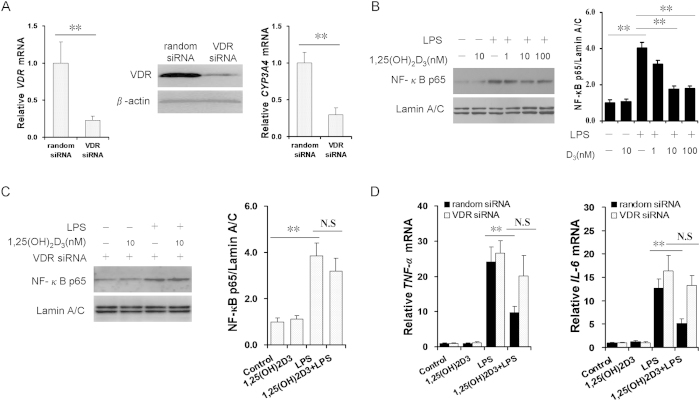
VitD3 inhibits placental NF-κB signaling in VDR-dependent manner. (**A**) VDR and CYP3A4 mRNAs were determined using real-time RT-PCR at 60 h after random or VDR siRNA transfection (n = 6). VDR protein was detected using immunoblots at 60 h after VDR siRNA transfection (n = 6). (**B**) Human JEG-3 cells were transfected with random siRNA (100 nM) and cultured with LPS (2.0 μg/ml) in presence or absence of 1,25-(OH)2D3 (1, 10, or 100 nM). Nuclear NF-κB p65 was detected using immunoblots at 2 h after LPS (n = 3). (**C**) Human JEG-3 cells were transfected with VDR siRNA (100 nM) and cultured with LPS (2.0 μg/ml) in presence or absence of 1,25-(OH)2D3 (10 nM). Nuclear NF-κB p65 was detected using immunoblots at 2 h after LPS (n = 3). (**D**) TNF-α and IL-6 mRNAs 2 h after LPS (n = 6). All data were expressed as means ± s.e.m. ^**^P < 0.01; N.S, No Significance.

To further explore the functional role of VDR in modulating placental NF-κB activity, siRNA was used to inhibit the expression of VDR in human JEG-3 cells. As expected, VDR mRNA and protein were down-regulated by 70% in VDR-siRNA-transfected JEG-3 cells. CYP3A4, a target of VDR, was down-regulated in VDR-siRNA-transfected JEG-3 cells (Fig. 9A). We then analyzed the effects of 1,25-(OH)2D3 on LPS-activated NF-κB signaling in human JEG-3 cells as well as in VDR-siRNA-transfected human JEG-3 cells. As expected, 1,25(OH)2D3 significantly attenuated LPS-induced translocation of NF-κB p65 in human JEG-3 cells in a dose-dependent manner (Fig. 9B). In contrast, 1,25(OH)2D3 had no effect on LPS-induced activation of NF-κB p65 in VDR-siRNA-transfected human JEG-3 cells (Fig. 9C). Similarly, 1,25(OH)2D3 did not inhibit LPS-induced *TNF-α* and *IL-6* up-regulation in VDR-siRNA-transfected human JEG-3 cells (Fig. 9D).

## Discussion

The present study investigated the effects of VitD3 supplementation during pregnancy on LPS-induced fetal IUGR. We found that pretreatment with VitD3 prevented LPS-induced fetal IUGR and demise. Several reports have demonstrated that maternal and placental inflammation plays a pivotal role in LPS-induced fetal IUGR and demise^13^,^17^. Indeed, the present study showed that serum TNF-α, IL-1β and IL-6 were significantly elevated in LPS-treated dams. Moreover, *tnf-α*, *il-1β*, and *il-6*, three inflammatory cytokines, and *mip-2*, *kc* and *mcp-1*, three chemokine genes, were markedly up-regulated in the placentas of LPS-treated mice. Interestingly, pretreatment with VitD3 obviously attenuated LPS-induced elevation of inflammatory cytokines in maternal serum. In addition, pretreatment with VitD3 inhibited LPS-induced up-regulation of inflammatory cytokines and chemokines in the placentas. These results support the hypothesis that VitD3 alleviates LPS-induced fetal IUGR and demise through its anti-inflammatory activity.

The mechanism through which VitD3 exerts its anti-inflammatory activity remains with debate. It has recently been reported that VitD3 represses LPS-induced MAPK p38 and Akt phosphorylation in macrophages^30^,^31^. Unexpectedly, the present study showed that VitD3 couldn’t inhibit LPS-induced placental p38 MAPK and ERK phosphorylation. Actually, pretreatment with VitD3 slightly aggravated LPS-induced placental Akt phosphorylation. Indeed, another study showed that VitD3-induced cell survival/proliferation through activating phosphorylation of Akt in human endothelial cells and skeletal muscle cells^32^,^33^. These studies indicated that the effect of VitD3 on regulating PI3K/Akt signaling pathway may be different in different cells or tissues. In addition, VitD3 pretreatment markedly repressed LPS-induced activation of placental NF-κB signaling. Generally, NF-κB subunits are retained in the cytoplasm through binding to the IκBα. When IκBα is phosphorylated, NF-κB subunits translocate to the nuclei to activate its downstream target genes^34^,^35^. The present study showed that VitD3 pretreatment significantly blocked LPS-induced placental translocation of NF-κB p65 subunit from cytoplasm to nuclei. Unexpectedly, VitD3 had little effect on LPS-induced placental IκBα phosphorylation. These results demonstrate that VitD3 inhibits placental NF-κB signaling independent of IκBα phosphorylation.

Several studies demonstrate that some nuclear receptors, such as small heterodimer partner (SHP), pregnane X receptor (PXR) and liver X receptor (LXR), inhibit NF-κB signaling in macrophages and intestinal epithelial cells^36^,^37^,^38^. On the other hand, it has long been known that LPS-activated toll-like receptor (TLR)4 and its downstream cytokines inhibit nuclear receptor signaling through activating NF-κB in human primary hepatocytes and intestinal epithelial cells^39^,^40^. VDR also is a nuclear receptor. Some reports demonstrate that vitamin D receptor (VDR) negatively regulates bacterial-induced intestinal NF-kappaB activation and attenuates response to infection^26^,^27^,^28^. Another study showed that VDR block TNFα-induced NF-κB activation and IL-6 up-regulation in HEK293 and RAW264.7 cells^29^. Indeed, VDR is highly expressed in human and rodent placentas^41^,^42^. Thus, the present study hypothesizes that VitD3 inhibits placental inflammation through reinforcing the interaction between VDR and NF-κB subunits. The important role of VDR on VitD3-mediated anti-inflammatory activity is demonstrated based on the following results: firstly, VitD3, which activated placental VDR, specifically repressed LPS-activated placental NF-κB and its downstream target genes. Secondly, LPS-evoked nuclear translocation of NF-κB p65 subunit was mainly distributed in trophoblast gaint cells of the labyrinth zone, where VitD3-induced nuclear translocation of VDR was observed. To further elucidate the mechanism through which VitD3-activated VDR inhibits placental inflammation, Co-IP was used to test physical association between placental VDR and NF-κB p65 subunit. As expected, VitD3 reinforced the interaction between placental VDR and NF-κB p65 subunit. This notion is further strengthened by observation that LPS-activated NF-κB antagonized VitD3-activated placental VDR signaling and its downstream target gene expression. Taken together, these results provide a mechanistic explanation for VitD3-mediated anti-inflammatory activity in placenta.

In the present study, VitD3 obviously attenuated LPS-induced placental translocation of NF-κB p65 subunit from cytoplasm to nuclei. Corresponding, LPS significantly inhibit VitD3-induced placental translocation of VDR from cytoplasm to nuclei. In addition, the combination stimulation with LPS and VDR will decrease the translocation of VDR and P65 into nucleus. These results further showed that the interaction between placental VDR and NF-κB p65 subunit is located in the cytoplasm.

VDR as a regulator of placental inflammation may have preventive and therapeutic implications. Indeed, several recent reports observed an association between maternal vitamin D deficiency during pregnancy and the risk of small-for-gestational-age (SGA)^43^,^44^,^45^. On the other hand, the levels of inflammatory cytokines were elevated in the cord blood from SGA infants^46^,^47^, suggesting an association between inflammation and IUGR. The present study showed that maternal VitD3 supplementation during pregnancy significantly alleviated LPS-induced placental inflammation and fetal IUGR. Therefore, VitD3 may be used as a potential protective agent for clinical prevention and therapy especially in high-risk situations in which the patients are infected with bacteria.

In summary, the present study investigated the effects of VitD3 supplementation during pregnancy on LPS-induced fetal IUGR and demise. Our results showed that pretreatment with VitD3 markedly alleviated LPS-induced fetal IUGR and demise through its anti-inflammatory activity. Moreover, we observed a mutual repression between VitD3-activated placental VDR and NF-κB p65 subunit. We demonstrate that VitD3 reinforces physical interaction between placental VDR and NF-κB p65 subunit. Finally, the further study demonstrates that VitD3 inhibits placental NF-κB signaling in VDR-dependent manner. These results provide a mechanistic explanation for VitD3-mediated anti-inflammatory activity. Overall, the present study provides evidence for roles of VDR as a key regulator of placental inflammation.

## Materials and Methods

### Chemicals and reagents

Lipopolysaccharide (*Escherichia coli* LPS, serotype 0127:B8) and Vitamin D3/Cholecalciferol were purchased from Sigma Chemical Co. (St. Louis, MO). Phosphor-p38 MAPK (pp38), p38, Phosphor-ERK1/2, phosphor-IκBα, IκBα, NF-κB p65, β-actin, α-tublin and Lamin A/C antibodies were from Santa Cruz Biotechnologies (Santa Cruz, CA). VDR antibodies were from Santa Cruz Biotechnologies (Santa Cruz, CA) and Abcam (Cambridge, MA). Phosphor-Akt (pAkt) and Akt antibodies were from Cell Signaling Technology (Beverley, MA, USA). Chemiluminescence (ECL) detection kit was from Pierce Biotechnology (Rockford, IL). TRI reagent was from Molecular Research Center, Inc (Cincinnati, Ohio). RNase-free DNase was from Promega Corporation (Madison, WI). All the other reagents were from Sigma or as indicated in the specified methods.

### Animals and treatments

The ICR mice (8~10 week-old; male mice: 28~30 g; female mice: 24~26 g) were purchased from Beijing Vital River whose foundation colonies were all introduced from Charles River Laboratories, Inc. The animals were allowed free access to food and water at all times and were maintained on a 12-h light/dark cycle in a controlled temperature (20–25 °C) and humidity (50 ± 5%) environment for a period of 1 week before use. All diets were purchased from TROPHIC Animal Feed High-tech Co. Ltd (Nantong, Jiangsu, China). For mating purposes, four females were housed overnight with two males starting at 9:00 P.M. Females were checked by 7:00 A.M. the next morning, and the presence of a vaginal plug was designated as gestational day (GD) 0. This study was approved by the Association of Laboratory Animal Sciences and the Center for Laboratory Animal Sciences at Anhui Medical University (Permit Number: 12-0005). All procedures on animals followed the Guide for the Care and Use of Laboratory Animals published by the US National Institutes of Health (NIH Publication No. 85-23, revised 1996). To investigate the effects of VitD3 on LPS-induced IUGR, all pregnant mice except controls were i.p. injected with LPS (100 μg/kg) daily from GD15 to GD17. In VitD3 + LPS group, pregnant mice were orally administered with VitD3 (25 μg/kg) daily from GD14 to GD17. The doses of VitD3 used in this study referred to others^48^. All animals were anesthetized with phenobarbital sodium (50 mg/kg) and sacrificed on GD18. Gravid uterine weights were recorded. For each litter, the number of live fetuses, dead fetuses and resorption sites were counted. Live fetuses in each litter were weighed and crown-rump length was measured.

### Cell culture and treatments

Human JEG-3 cells, a placental trophoblast-derived cell line, were grown in Nunc flasks in minimum essential medium (MEM, GIBCO) supplemented with 100 U/mL penicillin, 100 μg/mL streptomycin, 1.5 g/L NaHCO3, 0.11 g/L Sodium Pyruvate, and 10% (v/v) heat-inactivated FBS in a humidified chamber with 5% CO_2_/95% air at 37 °C. JEG-3 cells were seeded into 6-well culture plates at a density of 5 × 10^5^ cells/well and incubated for at least 12 h to allow them to adhere to the plates. After washing three times with medium, human JEG-3 cells were pre-incubated with different concentrations of 1,25-(OH)2D3 for 24 h. Human JEG-3 cells were incubated with LPS (2.0 μg/ml) for 2 h in the presence or absence of different concentrations of 1,25-(OH)2D3 (0, 1, 10 or 100 nM). The doses of 1,25-(OH)2D3 used in the present study referred to others^49^. The cells were washed with chilled PBS for three times and then harvested for real-time RT-PCR and immunoblots.

### Small interfering RNA (siRNA)

The expression of VDR levels was down-regulated in human JEG-3 cells using VDR small inhibitory RNA (siRNA) using standard techniques^50^. Human VDR gene was targeted by using ON-TARGET plus SMART pool siRNA which consists of four siRNA sequences: siRNA1, 5′ GCA ACC AAG ACU ACA AGU A 3′; siRNA2, 5′ GCG CAU CAU UGC CAU ACU G 3′; siRNA3, 5′ CCA ACA CAC UGC AGA CGU A 3′; siRNA4, 5′ GCA AUG AGA UCU CCU GAC U 3′. These pooled siRNAs were used at 100 nM with standard transfection protocol using lipofectamine 2000 (Invitrogen, Carlsbad, CA). Random siRNA (100 nM) was used as a control.

### Measurement of 25(OH)D

Pregnant mice non-fasting blood samples taken as part of routine antenatal care were collected and stored at –80 °C, with no further freeze-thaw cycles, until 25(OH)D measurement. Serum 25(OH)D was measured by Radioimmunoassay (RIA) with^125^ I labelled 25(OH)D as a tracer^45^,^51^, using a kit from Diasorin (DiaSorin Inc, Stillwater, MN, USA) following manufacturer’s instructions. Serum 25(OH)D is expressed as ng/ml.

### Real-time RT-PCR

Total RNA was extracted using TRI reagent. RNase-free DNase-treated total RNA (1.0 μg) was reverse-transcribed with AMV (Pregmega). Real-time RT-PCR was performed with a LightCycler 480 SYBR Green I kit (Roche Diagnostics GmbH) using gene-specific primers as listed in Table 2. The amplification reactions were carried out on a LightCycler 480 Instrument (Roche Diagnostics GmbH) with an initial hold step (95 °C for 5 minutes) and 50 cycles of a three-step PCR (95 °C for 15 seconds, 60 °C for 15 seconds, 72 °C for 30 seconds).

### Immunoblots (IB)

Total lysate from mouse placenta was prepared by homogenizing 50 mg placenta tissue in 300 μl lysis buffer (50 mM Tris-HCl, pH 7.4, 150 mM NaCl, 1 mM EDTA, 1% Triton X-100, 1% sodium deoxycholate, 0.1% sodium dodecylsylphate, 1 mM phenylmethylsulfonyl fluoride) supplemented with a cocktail of protease inhibitors (Roche). For nuclear protein extraction, total lysate from mouse placenta was suspended in hypotonic buffer and then kept on ice for 15 min. The suspension was then mixed with detergent and centrifuged for 30 s at 14,000 × g. The nuclear pellet obtained was resuspended in complete lysis buffer in the presence of the protease inhibitor cocktail, incubated for 30 min on ice, and centrifuged for 10 min at 14,000 × g. Protein concentrations were determined with the bicinchoninic acid (BCA) protein assay reagents (Pierce, Rockford, IL) according to manufacturer’s instructions. For immunoblots, same amount of protein (40~80 μg) was separated electrophoretically by SDS-PAGE and transferred to a polyvinylidene fluoride membrane. The membranes were incubated for 2 h with the following antibodies: NF-κB p65, VDR, pAKT/AKT, MyD88, pERK, pp38/p38 and pIκB. For total proteins, β-actin was used as a loading control. For nuclear protein, lamin A/C was used as a loading control. For cytoplasm protein, α-tublin was used as a loading control. After washes in DPBS containing 0.05% Tween-20 four times for 10 min each, the membranes were incubated with goat anti–rabbit IgG or goat anti–mouse antibody for 2 h. The membranes were then washed for four times in DPBS containing 0.05% Tween-20 for 10 min each, followed by signal development using an ECL detection kit.

### Electrophoretic mobility shift assay (EMSA)

Nuclear extracts were prepared from placenta tissue by the method of Deryckere and Gannon^52^. For EMSA, a biotin-labeled double-strand DNA probe containing the consensus DNA-binding sequence for NF-κB (5′-AGT TGA GGG GAC TTT CCC AGG C-3′ and 5′-GCC TGG GAA AGT CCC CTC AAC T-3′) was synthesized by Sangon Biological Technology (Shanghai, China). EMSA was performed with a LightShift Chemiluminescence electrophoretic mobility shift assay kit (Pierce Biotechnology, Inc., Rockford, IL). For competition assays, unlabeled NF-κB consensus oligonucleotides were used.

### Immunohistochemistry (IHC)

Paraffin-embedded placenta sections were deparaffinized and rehydrated in a graded ethanol series. After antigen retrieval and quenching of endogenous peroxidase, sections were incubated with either NF-κB p65 monoclonal antibody (1:200 dilution) or VDR monoclonal antibody (1:400 dilution, Abcam, ab3508) at 4 °C overnight. The color reaction was developed with HRP-linked polymer detection system and counterstaining with hematoxylin.

### Co-immunoprecipitation (Co-IP)

Placental cytosol proteins were prepared in a lysis buffer (0.6% Nonidet P-40, 0.5% sodium deoxycholate, 150 mM NaCl and 50 mM Tris-Hcl, pH 7.5) containing 0.1 mM vanadyl sulfate and protease inhibitors (0.5 mg/ml aprotinin, 0.5 mg/ml trans-epoxy succinyl-L-leucylamido-(4-guanidino)butane (E-64), 0.5 mg/ml pepstatin, 0.5 mg/ml bestatin, 10 mg/ml chymostatin, and 0.1 ng/ml leupeptin. Tissue lysates (300 μg) were precleared with protein A/G-agarose and then incubated with agarose-conjugated VDR antibody (Santa Cruz Biotechnology, Inc., Santa Cruz, CA) at 4 °C overnight. The precipitates were washed with cold RIPA buffer before immunoblots using a murine monoclonal NF-κB p65 antibody (Cascade Bioscience, Winchester, MA).

### Enzyme-linked immunosorbent assay (ELISA)

Commercial ELISA kits (R&D Systems, Abingdon, Oxon, UK) were used to measure mouse TNF-α, IL-1β and mouse IL-6 in maternal serum according to the manufacturer’s protocol.

### Statistical analysis

For animal experiments, the litter was considered the unit for statistical analysis among different groups. For fetal weight and crown-rump length, the means were calculated per litter and then averaged per group. All quantified data were expressed as means ± s.e.m. All statistical tests were two-sided using an alpha level of 0.05. ANOVA and the Student-Newmann-Keuls post hoc test were used to determine differences among different groups. Student *t* test was used to determine differences between two groups.

## Additional Information

**How to cite this article**: Chen, Y.-H. *et al.* Vitamin D3 inhibits lipopolysaccharide-induced placental inflammation through reinforcing interaction between vitamin D receptor and nuclear factor kappa B p65 subunit. *Sci. Rep.* **5**, 10871; doi: 10.1038/srep10871 (2015).

## Figures and Tables

**Table 1:** Fetal outcomes among different groups.

**Parameters**	**Control**	**VitD3**	**LPS**	**VitD3 + LPS**
No of pregnant mice (n)	15	15	14	15
No of abortion (n)	0	0	0	0
Resorptions per litter (n)	0.2 ± 0.1	0.3 ± 0.2	0.1 ± 0.1	0.3 ± 0.1
Dead fetuses per litter (n)	0.2 ± 0.1	0.2 ± 0.1	2.9 ± 0.8**	0.9 ± 0.2¶¶
Live fetuses per litter (n)	12.9 ± 1.2	12.5 ± 0.9	10.0 ± 1.2^*^	11.9 ± 0.7¶¶
Fetal weight (g)	1.42 ± 0.019	1.42 ± 0.012	1.19 ± 0.026**	1.34 ± 0.016¶¶
Crown-rump length (cm)	2.50 ± 0.012	2.50 ± 0.007	2.33 ± 0.018**	2.45 ± 0.016¶¶
Average placental weight (g)	0.101 ± 0.002	0.102 ± 0.004	0.084 ± 0.002**	0.094 ± 0.002¶¶

^a^The number of dead or live fetuses per litter in dams that completed the pregnancy. All data were expressed as means ± s.e.m.

^*^P < 0.05, ^**^P < 0.01 as compared with Control; ^¶¶^P < 0.01 as compared with LPS.

**Table 2:** Oligonucleotide sequences and size of primers.

**Genes**	**Sequences**	**Sizes (bp)**	Species
*gapdh*	Forward: 5′- ACCCCAGCAAGGACACTGAGCAAG -3′Reverse: 5′- GGCCCCTCCTGTTATTATGGGGGT -3′	109	mouse
*tnf-α*	Forward: 5′- CCCTCCTGGCCAACGGCATG -3′Reverse: 5′- TCGGGGCAGCCTTGTCCCTT -3′	109	mouse
*il-6*	Forward: 5′- AGACAAAGCCAGAGTCCTTCAGAGA -3′Reverse: 5′- GCCACTCCTTCTGTGACTCCAGC -3′	146	mouse
*il-1β*	Forward: 5′- GCCTCGTGCTGTCGGACCCATAT-3′Reverse: 5′- TCCTTTGAGGCCCAAGGCCACA -3′	143	mouse
*mip-2*	Forward: 5′- TTGCCTTGACCCTGAAGCCCCC -3′Reverse: 5′- GGCACATCAGGTACGATCCAGGC -3′	175	mouse
*kc*	Forward: 5′-ACTCAAGAATGGTCGCGAGG-3′Reverse: 5′-GTGCCATCAGAGCAGTCTGT-3′	123	mouse
*mcp-1*	Forward: 5′- GGCTGGAGAGCTACAAGAGG-3′Reverse: 5′-GGTCAGCACAGACCTCTCTC-3′	93	mouse
*tlr4*	Forward: 5′- TCAGCAAAGTCCCTGATGACATTCC-3′Reverse: 5′- AGAGGTGGTGTAAGCCATGCCA-3′	180	mouse
*cyp24a1*	Forward: 5′- CCCCAAGTGCAACAGAGACT-3′Reverse: 5′- CCGAGTTGTGAATGGCACAC-3′	153	mouse
*p21*	Forward: 5′- TAAGGACGTCCCACTTTGCC-3′Reverse: 5′- AAAGTTCCACCGTTCTCGGG-3′	197	mouse
*β-actin*	Forward: 5′- TCCCTGGAGAAGAGCTACGA-3′Reverse: 5′- AGCACTGTGTTGGCGTACAG-3′	194	human
*VDR*	Forward: 5′- ACCAGAAGCCTTTGGGTCTG-3′Reverse: 5′- CGTTCCGGTCAAAGTCTCCA-3′	146	human
*CYP3A4*	Forward: 5′- TGCACATAGCCCAGCAAAGA-3′Reverse: 5′- ATAGAGGAGCACCAGGCTGA-3′	145	human
*TNF-α*	Forward: 5′- CACCACTTCGAAACCTGGGA-3′Reverse: 5′- TGTAGGCCCCAGTGAGTTCT-3′	105	human
*IL-6*	Forward: 5′- CTTCGGTCCAGTTGCCTTCT-3′Reverse: 5′- GATGCCGTCGAGGATGTACC-3′	169	human
